# A CXCR3-Activating Peptide Increases Tear Break Up Time and Corrects Corneal haze in a Rabbit Model of Environmental Dry Eye

**DOI:** 10.26502/fjppr.094

**Published:** 2024-04-17

**Authors:** Alan Wells, Yadong Wang, Hanshuang Shao, Peri Sohnen, Shivalingappa K. Swamynathan

**Affiliations:** 1 Department of Pathology, University of Pittsburgh, Pittsburgh, PA, USA; 2 McGowan Institute of Regenerative Medicine, University of Pittsburgh, Pittsburgh, PA, USA; 3 Department of Ophthalmology, University of Pittsburgh, Pittsburgh, PA, USA; 4 Fox Center for Vision Restoration, University of Pittsburgh, Pittsburgh, PA, USA; 5 Department of Cell Biology, University of Pittsburgh, Pittsburgh, PA, USA; 6 Department of Bioengineering, Cornell University, Ithaca, NY, USA; 7 Department of Ophthalmology, University of South Florida, Tampa, FL, USA

**Keywords:** CXCL10, CXCR3, dry eye disease, goblet cells, inflammation, keratoconjunctivitis sicca, meibomian glands

## Abstract

**Purpose:**

Environmentally-triggered dry eye disease (DED) or keratoconjunctivitis sicca (KCS), which constitutes the majority of DED cases, currently is palliatively treated with aqueous replacement solutions that do not target the dysfunction of the mucin and lipid components of tears. We tested whether a peptide that increased goblet cell numbers in a model of scleral chemical injury would also improve tear quality in environmental DED.

**Methods:**

Environmental DED was established by exposing New Zealand white rabbits (8 per group, female) to 20% humidity with rapid air replacement and b.i.d. atropine sulfate eyedrops for 3 weeks prior to test article administration; this continued for the subsequent 3 weeks of testing. Animals were dosed by (A) saline, (B) b.i.d. eyedrop of peptide in saline, (C) b.i.d. eyedrop of peptide in coacervate, or (D) weekly subconjunctival injection of peptide. In vitro, human conjunctival epithelial cells (HCjE) were exposed to TNFα in the presence or absence of peptide to determine inflammatory responsiveness.

**Results:**

The environmental DED was established with both Schirmer and TBUT being reduced at the start of test article; these levels were maintained as low through the testing period. All three treatment regimens increased TBUT approximately 3x to levels greater than prior to desiccation (P < 0.01), with little effect on Schirmer. Corneal haze was present in all eyes after induction, and completely reversed in 36 of 48 eyes across the treatments (P < 0.05). Co-treatment of HCjE with peptide reduced the production of TNFα in response to an inflammatory stimulus.

**Conclusions:**

The treatment of environmental DED/KCS with a peptide that activates CXCR3 improved tear quality and reversed corneal pathology by promoting tear stability and likely dampening the corneal inflammation, while not affecting aqueous volume of the tears.

## Introduction

Dry eye disease (DED), technically known as keratoconjunctivitis sicca (KCS), is a syndrome with a number of causes [1–3]. The basal phenotype of insufficient tear coverage of the sclera is often treated palliatively with physical replacement of tears. The only treatments that target a cause of DED are those that ameliorate auto-immune disease related inflammation [4], however these immunosuppressive agents are germane against only about 1 in 10 cases of DED. This leaves the vast majority of cases with only temporary symptomatic relief.

Regardless of the mechanistic cause, the tissue manifestations of DED converge on damage to the sclera of the eye with ongoing chronic inflammation [2, 5]. The goblet cells malfunction and are eventually lost due to the non-specific inflammatory damage. This leads to a decrease in the mucin layer of the tears, with resultant inability of the aqueous layer to adhere to the scleral surface [1].

Serendipitously, we found that activators of the CXCR3 receptor can provide for goblet cell rescue in a rabbit model of toxic dry eye [6]. After trabeculectomy, subconjunctival injection of a peptide activator of CXCR3 led to an increase in number of goblet cells to levels higher than prior to surgery. Even in the face of mitomycin C, goblet cells rebounded to pre-surgical levels. This is intriguing due to the recent focus on the centrality of mucins in DED [5, 7]. Based on these findings, we investigated whether CXCR3 activators could alleviate DED in causes in addition to surgical and toxic injury. Herein, we report that a peptide activator of CXCR3 was restored mucin/lipid functioning in a rabbit model of environmental DED.

## 2. Methods

### 2.1 Materials

The peptide is a 22-mer (OH-PESKAIKNLLKAVHKEMSKRSP-NH2) manufactured by PolyPeptide Inc (San Diego, CA, USA) to >95% purity as a lyophilized powder. This was delivered in three different formulations. For subconjunctival injection, 3ug were dissolved in 100ul of sterile PBS. For eyedrops, 10ug were dissolved in 50ul of sterile PBS). For the slow release formulation, 10ug were dissolved in 50ul of coacervate. The control was 50ul of sterile PBS. The coacervate is a Heparin and PEAD (1:3.6) mixture with each component at a 10mg/mL concentration in water [8, 9]. The peptide was added at a 1:10 dilution in PBS to the final concentration.

### 2.2 Study Design

The environmental dry eye model was tested under contract at Absorptions Systems (San Diego, CA, USA). This CRO follows the ARVO guidelines for animal research, and the study was approved by the ASC IACUC prior to initiation of the study. ASC is AAALAC-accredited, in compliance with ASC’s Animal Welfare Assurance (D16–00645 [A4282–01]) filed with the NIH.

DED is more common in women; we used female New Zealand White Rabbits (8 per group), at 125–175 days old (3–5 kg). Animals were dosed with Atropine Sulfate (1%) in both eyes (OU) 2x daily (BID) for 3 weeks prior to test article administration to induce KCS. Humidity was maintained at ~20% throughout the study to promote eye dryness. Both were continued for all animals until study completion. The study was conducted as outlined in Table 1. This was done in two staggers with half the rabbits in each group (4) being treated in each stagger.

### 2. 3 TNFα-mediated inflammation

Transformed human conjunctival epithelial cells (HCjE; received from Dr. Ilene Gipson, Harvard University) were seeded at 40,000 cells per well in a 96 well flat bottom plate. After overnight adhesion in Keratinocyte-Serum Free Media (cK-SFM), the cells were washed and exposed to starvation media (SFM) for a further 24 hours. At this time the cells were exposed to TNFα (5ng/ml) in the presence or absence of peptide (200ng/ml). After 4 hours the cells were harvested and tested for TNFα mRNA levels with GAPDH mRNA as the control for equalization.

## 3. Results

**Figure 1:**
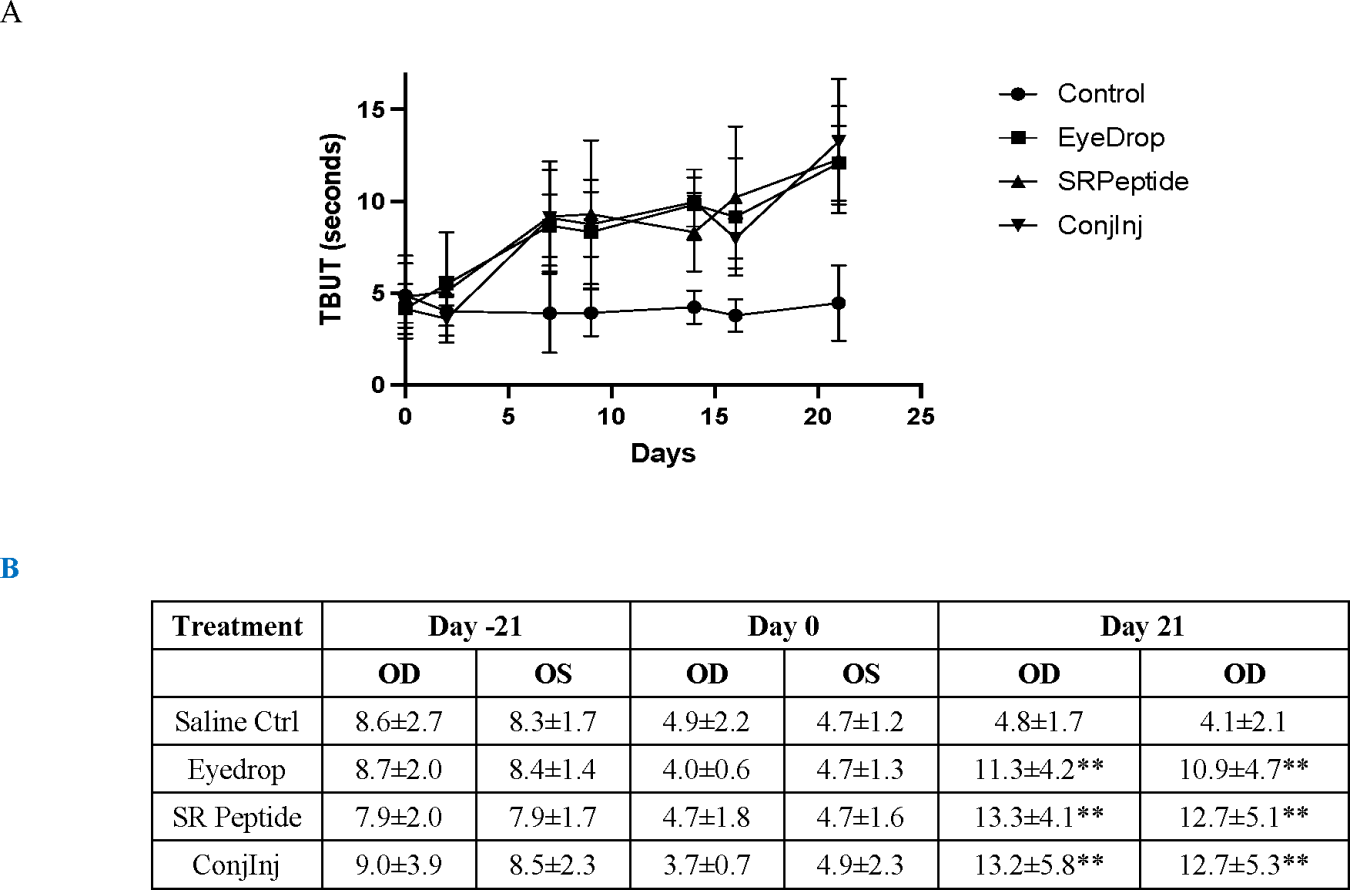
Increase in TBUT upon treatment of rabbits. Rabbits (8 per group) were treated with the peptide weekly in subconjunctival injection formulation, or twice per day in saline eyedrops or in a coacervate formulation. The control was saline eyedrops twice per day in both eyes. DED was induced for 21 days prior to treatments. On days 2, 7, 9, 14, 16, and 21 the eyes were assessed for TBUT. Presented are averages, with all treatment groups being statistically greater than saline control by day 7 (A). (B) Induction reduced the TBUT in all eyes, with all treatments restoring TBUT to above baseline (**p < 0.01 using t-Test comparisons) while saline treatment did not increase TBUT.

The main measurements relevant to tear function were the Schirmer Tear Test (STT) and TBUT times. The induction and maintenance of desiccation-induced dry eye led to a > 30% reduction in STT (Table 2), and ~ 50% reduction in TBUT (Figure 1). For the STT, the baseline values of 12–15mm were reduced minimally to 9–12mm after induction, and tended to drift lower during treatment to 8–11mm, regardless of the delivery mode (Table 2). Thus, the amount of aqueous tear was uncorrected by the CXCR3 activating peptide. TBUT was altered much more by the induction and treatments. Initial TBUT of 8–9s were reduced to half (3.5–5s) after induction and prior to treatment (Figure 1). TBUT remained low in the saline treated eyes but rebounded to baseline in the first week of treatment of all three deliveries (Figure 1). Interestingly in all three treatment groups, the TBUT exceeded baseline by the end of week 3.

The increase in the TBUT combined with no effect on STT suggested that either the mucin or lipid layer was altered, in accordance with the earlier findings on an increase in goblet cells after toxic (mitomycin-C) injury [6]. Thus, we asked whether the peptide altered the goblet cells. Histopathological examination of the enucleated globes at the end of the study (day 21 of treatment) found that goblet cell density was similarly high in all treatment and control groups, suggesting that the induction of dry eye was not of sufficient duration to affect goblet cell numbers in this study (data not shown).

Still, the treatments did alleviate the desiccation-induced corneal pathology. All 64 eyes displayed corneal haze at the end of the three-week induction period. At the end of the three-week test article administration the control group still had corneal pathology in all 16 eyes. However, the number of affected eyes were reduced after treatment with eyedrops (4 of 16 eyes showing pathology), slow-release gel formulation (2 of 16) and subconjunctival injection (6 of 16). All treatments were statistically different from the saline-treated control (at P < 0.05).

**Figure 2:**
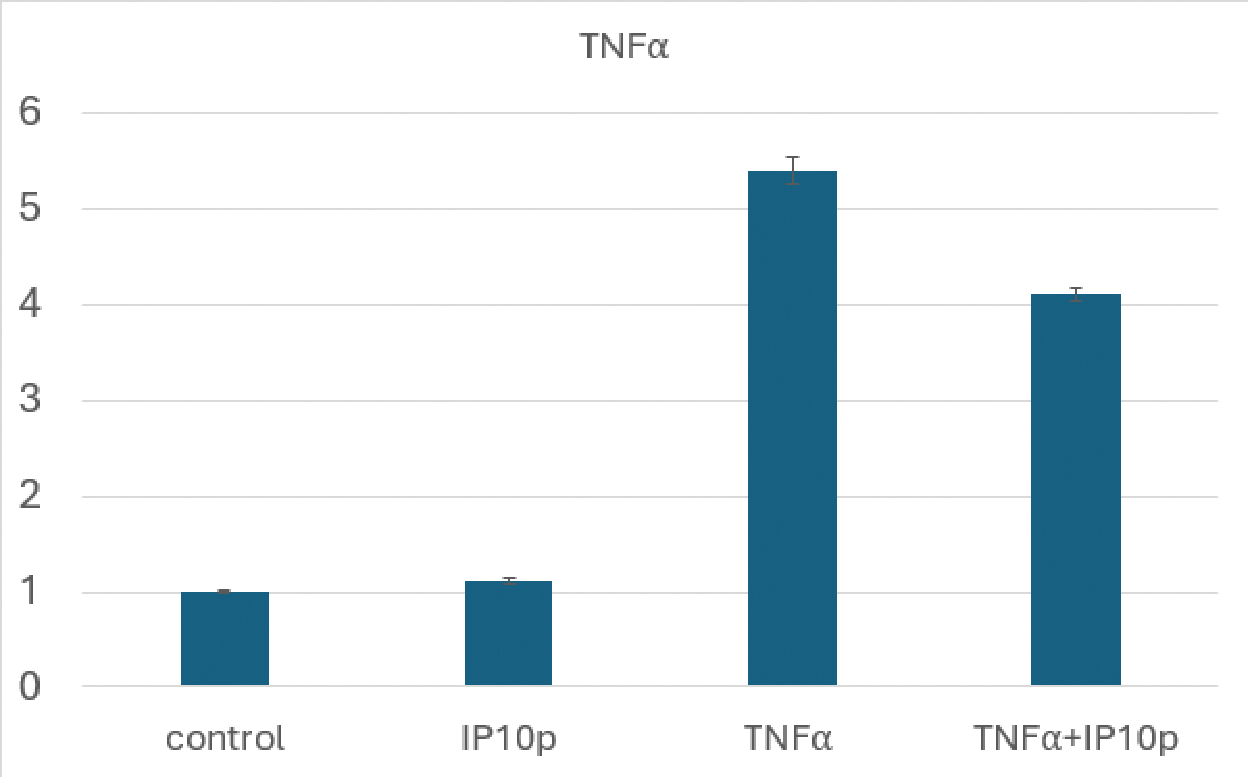
Decreased TNFα production in the presence of peptide. HCjE were exposed to TNFα (5ng/ml) in the presence or absence of peptide (200ng/ml) for 4 hours prior to harvesting and qRT-PCR. As seen, even in this in vitro challenge on stiff plastic, peptide reduced TNFα mRNA levels by about a quarter. Shown is the mean ± s.d. of three experiments each in triplicate; P < 0.01 comparing TNFα + IP10p to TNFα.

An initial in vitro study to discern the underlying mechanism of action focused on the anti-inflammatory nature of CXCR3 signaling [10]. We examined the inflammatory potential of conjunctival epithelial cells (HCjE) that are involved during flares in DED [11]. We found that the CXCR3-activating peptide blocked the stimulation of TNFα mRNA in these cells (Figure 2).

## Discussion

Dry eye disease results from a plethora of insults to the surface of the eye [1–3], with only a small number of auto-immune conditions having disease modifying treatments (mainly non-specific immune suppression) [4, 12]. The vast majority of the cases are treated symptomatically. When the insult is not a singular episode such as a toxic substance or ocular surgery [13], this leads to a chronic condition. As environmental causes are impractical to avoid for most patients, new treatments that have the potential to modify the course of the disease are needed.

The pathophysiologies of DED eventually converge on damage to the mucin-producing goblet cells [1, 7] that in turn leads to the inability of tears to adhere to the ocular surface. To improve goblet cell functioning and survival, we serendipitously found that a peptide activator of CXCR3 increased the number of goblet cells after ocular surface insult during trabeculectomy [6]. Herein we found that the same stimulus could reverse dry eye due to environmental insult.

A second line of inquiry looked at the propagation of desiccation-induced inflammation from conjunctival epithelial cells. As the ocular surface is exposed during chronic DED, the damage is compounded by an inflammatory response of the ocular surface epithelial cells [5, 11]. As TNFα is central to both the initial inflammation and the conjunctival epithelial cell propagation [11], we asked whether this was dampened by exposure to the CXCR3-activating peptide. Our initial studies found that even in vitro, the peptide could blunt the inflammatory feed-forward mechanism. While these studies will be needed to be translated into animal models and later tested in human tears, it points the way towards how activation of the pleiotropic CXCR3 receptor can function at multiple levels to restore the homeostasis of the ocular surface.

There are a number of caveats in translating these findings to the human condition, not the least being that rabbit eyes have high densities of goblet cells and robust TBUT. Thus, whether such enhanced functioning will transfer to the human condition or to similar extent will require trials in patients. Second, loss of the aqueous component of tears may remain a barrier in many patients for whom tear volume is a challenge. In such cases a combinatorial approach would make intuitive sense. For situations in which ongoing auto-immune or allergic inflammation initiates the ocular surface damage, just supporting the goblet cells is unlikely to be disease modifying and also require combinatorial therapies. Despite these concerns, it is only upon testing in patients can we address whether this approach represents a new quiver in our armamentarium against dry eye disease.

## Figures and Tables

**Table 1:** Study design

Group	N	Treatment	Route & Regimen	Dose Volume (μL/eye)	Slit Lamp Exams	Fluorescein TBUT; Slit Lamp Photos; Fluorescein Staining	Schirmer Tear Test
**1**	8	Control	Topical BID starting on	50 μL sterile PBS	Baseline (prior to induction), once during induction but prior to TA admin and once weekly after Day 1 until completion of treatment phase	Prescreen++,Baseline (prior to TA administration), and twice weekly after Day 1 until the completion of treatment phase	Prescreen, twice weekly (during induction)**, Baseline (prior to TA administration) and after Day 1 until the completion of treatment phase
**2**	8	Topical Drops	Day 1 (OU)	50 μL			
				10 μg Peptide			
**3**	8	Topical Gel		50 μL			
				10 μg Peptide			
**4**	8	Sub-Conjunctival Injection	Single subconjunctival injection (OU) on Days 1, 7,	100 μL ***			
			and 14	3 μg Peptide			

Schirmer Tear Tests (STT) were conducted to measure tear volume by standard methods. Tear breakup time (TBUT) was used to assess evaporative dry eye by standard methods. The investigators performing all TBUT and STT evaluations were blinded to the treatment groups.

**Table 2:** STT values prior to induction, prior to treatment and after treatment

Treatment	Day -21		Day 0		Day 21		P Value
	OD	OS	OD	OS	OD	OD	
Saline Ctrl	15.5±3.2	13.1±2.9	12.1±3.6	9.0±2.9	8.4±2.2	8.3±3.0	n.s.
Eyedrop	14.6±5.6	14.0±2.8	11.3±3.3	9.9±3.7	9.9±3.9	7.9±2.0	n.s.
SR Peptide	14.6±3.3	13.0±4.1	9.8±3.4	9.3±3.2	9.4±2.9	8.4±1.8	n.s.
ConjInj	12.6±2.8	12.5±2.7	10.3±2.5	8.8±2.7	11.0±1.9	9.8±2.0	n.s.

Shown are mean ± s.d. for 8 eyes in each column P values are day 21 versus at induction (Day 0). The saline control was significantly reduced from prior to induction but unchanged from prior to treatment.

## Data Availability

All relevant data are presented in this manuscript. Individual animal data are available from the authors.
